# Inhibition of Tat activity by the HEXIM1 protein

**DOI:** 10.1186/1742-4690-2-42

**Published:** 2005-07-02

**Authors:** Alessandro Fraldi, Francesca Varrone, Giuliana Napolitano, Annemieke A Michels, Barbara Majello, Olivier Bensaude, Luigi Lania

**Affiliations:** 1 Department of Structural and Functional Biology, University of Naples 'Federico II', Naples, Italy; 2 UMR 8541 CNRS, Ecole Normale Supérieure, Laboratoire de Régulation de l'Expression Génétique, Paris, France; 3 Telethon Institute of Genetics and Medicine (TIGEM) Naples, Italy

## Abstract

**Background:**

The positive transcription elongation factor b (P-TEFb) composed by CDK9/CyclinT1 subunits is a dedicated co-factor of HIV transcriptional transactivator Tat protein. Transcription driven by the long terminal repeat (LTR) of HIV involves formation of a quaternary complex between P-TEFb, Tat and the TAR element. This recruitment is necessary to enhance the processivity of RNA Pol II from the HIV-1 5' LTR promoter. The activity of P-TEFb is regulated in vivo and in vitro by the HEXIM1/7SK snRNA ribonucleic-protein complex.

**Results:**

Here we report that Tat transactivation is effectively inhibited by co-expression of HEXIM1 or its paralog HEXIM2. HEXIM1 expression specifically represses transcription mediated by the direct activation of P-TEFb through artificial recruitment of GAL4-CycT1. Using appropriate HEXIM1 mutants we determined that effective Tat-inhibition entails the 7SK snRNA basic recognition motif as well as the C-terminus region required for interaction with cyclin T1. Enhanced expression of HEXIM1 protein modestly affects P-TEFb activity, suggesting that HEXIM1-mediated repression of Tat activity is not due to a global inhibition of cellular transcription.

**Conclusion:**

These results point to a pivotal role of P-TEFb for Tat's optimal transcription activity and suggest that cellular proteins that regulate P-TEFb activity might exert profound effects on Tat function *in vivo*.

## Background

The positive transcription elongation factor b (P-TEFb) composed by CDK9/CyclinT1, has emerged as a significant co-factor of the HIV Tat protein. P-TEFb complex has been shown to associate with and phosphorylate the carboxyl-terminal domain (CTD) of RNA pol II, thereby enhancing elongation of transcription [1-3]. Tat protein binds an uracil containing bulge within the stem-loop secondary structure of the Tat-activated region (TAR-RNA) in HIV-1 transcripts [4-6]. Tat functions as an elongation factor and stabilizes the synthesis of full-length viral mRNAs by preventing premature termination by the TAR-RNA stem-loop. Physical and functional interactions between Tat and P-TEFb have been well documented [7,8]. Tat binds to P-TEFb by direct interaction with the human cyclinT1, and the critical residues required for interaction have been delineated [9,10]. The current model for recruitment of P-TEFb to the LTR, predicts the formation of the Tat-P-TEFb complex, which efficiently binds TAR, allowing CDK9 to phosphorylate the CTD of RNAPII, thereby, enhances processivity of the polymerase to produce full-length mRNAs [3,7-10].

Like other CDKs, the P-TEFb activity is regulated by a dedicated inhibitor. Two different P-TEFb complexes exist in vivo [11,12]. The active complex is composed of two subunits, the CDK9 and its regulatory partners cyclinT1 or T2. In addition, a larger inactive complex has been identified, which comprises of four subunits, CDK9, cyclinT1 or T2, the abundant small nuclear RNA 7SK and the HEXIM1 protein [13-17]. It has been recently shown that HEXIM1 has the inherent ability to associate with cyclin T1 and binding of 7SK snRNA turns the HEXIM1 into a P-TEFb inhibitor [15-17]. The relative presence of core and inactive P-TEFb complexes changes rapidly in vivo [11,12]. Several stress-inducing agents trigger dissociation of the inactive P-TEFb complex and subsequent accumulation of kinase active P-TEFb [11]. Thus, the 7SK-HEXIM1 ribonucleic complex represents a new type of CDK inhibitor that contributes to regulation of gene transcription. A further level of complexity of this system comes from the recent identification of HEXIM2, a HEXIM1 paralog, which regulates P-TEFb similarly as HEXIM1 through association with 7SK RNA [18,19].

It has been showed that Tat binds exclusively to the active P-TEFb complex [13]. Thus the presence of HEXIM1/7SK snRNA in P-TEFb complexes prevents Tat binding. Since the association between 7SK RNA/HEXIM1 and P-TEFb appears to compete with binding of Tat to cyclinT1, we have speculated that the TAR RNA/Tat system may compete with the cellular 7SK snRNA/HEXIM1 system in the recruitment of the active P-TEFb complex [13]. Accordingly, it has been shown that over-expression of HEXIM1 represses Tat function [14,17].

We show here that HEXIM1, or its paralog HEXIM2, inhibits Tat trans-activation of HIV-LTR driven gene expression, and more importantly, we demonstrated the role of the 7SK snRNA recognition motif as well as the binding to cyclin T1 as crucial elements for efficient Tat inhibition.

## Results

### Tat activity is inhibited by HEXIM1

**Figure 1:**
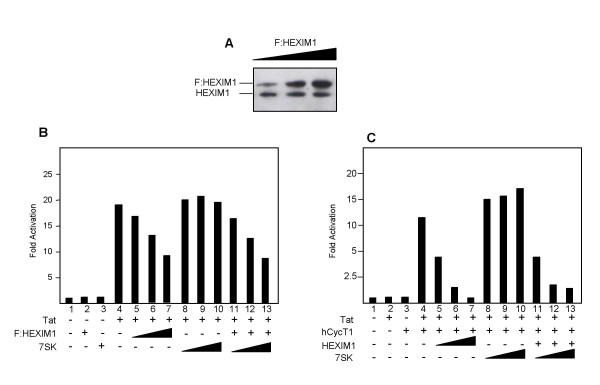
Overexpression of HEXIM1 protein represses Tat transactivation. Panel A, Increasing amounts (10, 100 and 500 ng) of Flag-taggeted HEXIM1 were transfected into 293, cellular extracts were prepared at 48 hr after transfection and the relative levels on endogenous and exogenous HEXIM1 proteins were visualized by immunoblotting with anti-HEXIM1 antibody. Panel B, the HIV-Luc reporter (50 ng) was transfected into 293 cells in the presence of pSV-tat (50 ng) along with increasing (10, 100 and 500 ng) amounts of F:HEXIM1 and 7SK RNA (10, 100 and 500 ng), as indicated. Panel C, HEXIM1 decreases the co-operative effect of CycT1 on Tat activation in rodent cells. Chinese hamster ovary cells (CHO) were transfected with the HIV-LTR-Luc reporter (50 ng) in the presence of pSV-Tat (100 ng), lane 1, and together with CMV-hCycT1 (200 ng), lane 2, in the presence of increasing amounts of F:HEXIM1 and 7SK RNA as in panel B. Each histogram bar represents the mean of at least three independent transfections after normalization to Renilla luciferase activity to correct for transfection efficiency with the activity of the reporter without effect set to one. Standard deviations were less than 10%.

Tat activity involves direct interaction with CDK9/CyclinT1 (P-TEFb) complex. However, two major P-TEFb-containing complexes exits in human cells [11,12]. One is active and restricted to CDK9 and cyclin T, the other is inactive and it contains HEXIM1 or 2 and 7SK snRNA in addition to P-TEFb [15,17]. We have previously shown that Tat interacts only with the active P-TEFb complex [13]. Because the two complexes are in rapid exchange, we sought to determine the functional consequences of the over-expression of HEXIM1 and 7SK snRNA on HIV-1 LTR-driven gene transcription. To this end we performed transient transfections in human 293 cells using the HIV-LTR-Luc reporter in the presence of increasing amounts of Flag-taggeted HEXIM1 and 7SK snRNA, respectively. Dose-dependent expression of F:HEXIM1 was monitored by immunoblotting with anti-HEXIM1 antibody (Fig. 1 panel A). As presented in Fig. 1B, we found that basal transcription from the LTR sequences was unaffected by the presence of F:HEXIM1 or 7SK RNA. In contrast, Tat-mediated transactivation of the HIV-1 LTR was inhibited by the over-expression of F:HEXIM1 in a dose-dependent manner. Ectopic expression of 7SK RNA did not significantly affected HIV-LTR-Luc expression either alone or in combination with F:HEXIM1. Thus, it appears that HEXIM1 is able to repress Tat-mediated activation. To further substantiate the inhibitory function of HEXIM1 we sought to extend our analysis using the murine CHO cells. Tat protein is a potent activator of HIV-1 LTR transcription in primate cells but only poorly functional in rodent cells [6,7]. However, Tat-mediated activation can be rescued by enforced expression of human cyclin T1 [6,7]. As presented in Fig. 1C we found that, while hCycT1 rescued Tat function, ectopic expression of HEXIM1 effectively inhibits Tat activity. Most importantly, Tat enhancement mediated by hCycT1 was effectively abrogated by co-expression of HEXIM1 in a dose-dependent manner. Finally, like in human cells, ectopic expression of 7SK snRNA did not have any significant effect on Tat activity.

**Figure 2:**
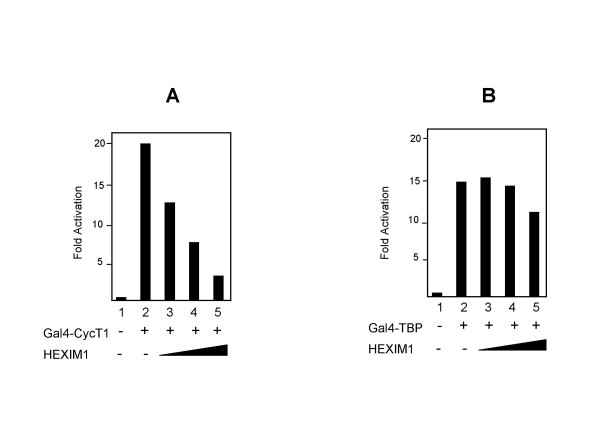
HEXIM1 represses GAL4-CycT1-mediated activation. Human 293 cells were transfected with 50 ng of G5-HIV Luc reporter DNA alone (lane 1) or in the presence of GAL4-expression plasmid DNA (200 ng), as indicated. The presence of the cotransfected F:HEXIM1 (10, 100 and 500 ng) is indicated. Each histogram bar represents the mean of three independent transfections after normalization to Renilla luciferase activity. The results are presented as described in figure 1.

The results reported above suggested that ectopic expression of HEXIM1 inhibits Tat activity. A large number of evidences indicate that Tat-transactivation is mainly due to the recruitment of the cellular complex P-TEFb to the LTR, causing phosphorylation of the RNAPII CTD [1,6-10]. Accordingly, we and others have previously showed that artificial recruitment of P-TEFb to the HIV-1 promoter is sufficient to activate the HIV-1 promoter in the absence of Tat [20,21]. We sought to determine the consequences of ectopically expressed F:HEXIM1 on P-TEFb induced transcription in the absence of Tat. We showed that direct recruitment of CyclinT1 to a promoter template by fusion to the GAL4 DNA binding domain, activates transcription from an HIV-1 LTR (G5HIV-Luc) reporter bearing GAL4 sites [20]. Human 293 cells were transfected with the G5HIV-Luc reporter along with GAL4-fusion expression vectors in the presence of F:HEXIM1. As shown in Fig. 2A, we found that GAL4-CycT1 effectively activates transcription from the HIV-1 LTR reporter, and co-expression of F:HEXIM1 resulted in a robust dose-dependent inhibition. The specific effect of HEXIM1 expression was highlighted by the results shown in Fig. 2B. G5HIV-Luc reporter was activated by co-expression of a GAL4-TBP, and such activation was largely unaffected by co-expression of HEXIM1. Thus, it appears that while HEXIM1 represses P-TEFb activity, enforced expression of this protein does not have significant effects on TBP-mediated basal transcription.

### Definition of the HEXIM1 regulatory domains involved in repression

**Figure 3:**
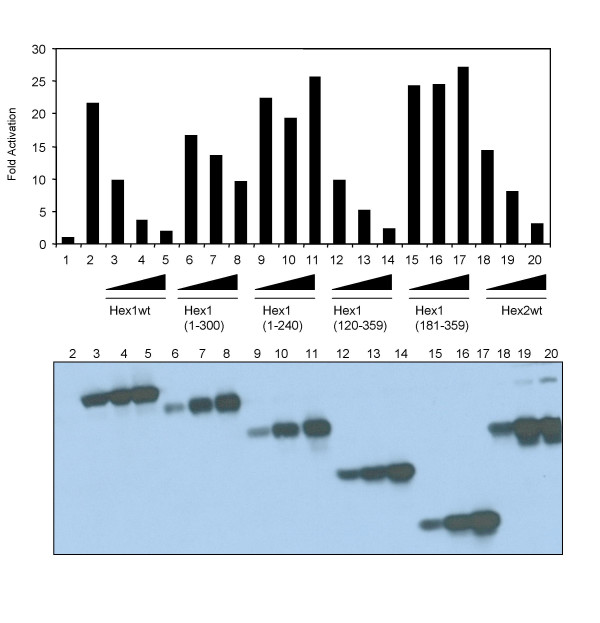
HEXIM1 regulatory domains involved in repression. Human 293 cells were transfected with 50 ng of G5-HIV Luc reporter DNA alone (lane 1) or in the presence of 50 ng of pSV-Tat (lanes 2–20). The presence of increasing amounts (10, 100 and 500 ng) F:HEXIM1 wild-type (lanes 3–5), various deletion mutants (lanes 6–17) and F:HEXIM2 wt(18–20) are indicated, respectively. On the bottom, it is shown the western-blot of whole cells extracts from transfected cells probed with anti-Flag antibody from the indicated co-transfections. The results presented are from a single experiment after normalization to Renilla luciferase activity with the activity of the reporter without effect set to one. This experiment was performed three times with similar results.

To investigate the structural determinants of HEXIM1 protein in repression, the activity of Gal4-CycT1 on G5HIV-Luc was monitored in the presence of co-transfected Flag-tagged deletion mutants of HEXIM1. We found that removal of the C-terminal amino acids affected the inhibition as shown by the HEXIM1 (1–300) and (1–240) mutants (Figure 3 lanes 6–8 and 9–11). In contrast, removal of the 119 N-terminal amino acids of HEXIM1 (120–359) did not abolished inhibition (lanes 12–14). However, further deletion of the N-terminal amino acids (181–359) completely abolished the inhibitory effect (lanes 15–17). Thus, HEXIM1-mediated repression required the presence of the C-terminal domain (300–359aa) as well as a central region between residues 120 and 181. Finally, we found that HEXIM2, which like HEXIM1, associates and inhibits P-TEFb activity, represses Gal4-CycT1 activation in a dose dependent manner (lanes 18–20).

**Figure 4:**
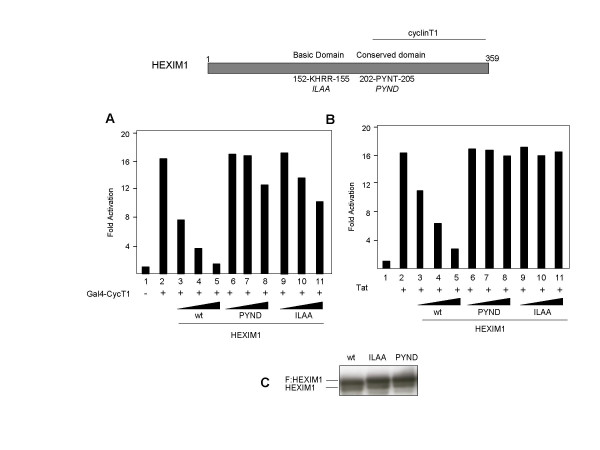
On top the relevant HEXIM1 functional domains are depicted. Position of the point mutants ILAA and PYND are indicated. G5-HIVLuc reporter (50 ng) was transfected into 293 cells along with Gal4-CycT1 (200 ng) Panel A, or pSV-Tat (50 ng) panel B along with increasing amounts of Flag:HEXIM1 wilt type and mutants (10, 100 and 500 ng) as indicated. Each histogram bar represents the mean of three independent transfections after normalization to Renilla luciferase activity. The results are presented as described in figure 1. Panel C, western-blot with anti-HEXIM1 antibody demonstrated that the HEXIM1 effectors were expressed at comparable levels.

We have recently reported that the HEXIM1 C-terminal domain (181–359) is involved in the binding to P-TEFb through direct interaction with the cyclin-box of cyclinT1 [15], and the evolutionarily conserved motif (PYNT aa 202–205) is important for such interaction. The PYND point mutant is impaired in repression and binding either P-TEFb or 7SK RNA in vivo, albeit it retains the ability to bind 7SK in vitro. In addition, we determined that HEXIM1 binds 7SK snRNA directly and the RNA-recognition motif (KHRR) was identified in the central region of the protein (aa 152–155). In fact, the HEXIM1-ILAA mutant fails to interact in vivo and in vitro with 7SK snRNA [15]. To test the importance of these motifs in HEXIM1-mediated repression of Tat activity, HEXIM1 point mutants were co-transfected in 293 cells along with Tat or Gal4-CycT1, respectively. As shown in Figure 4, unlike wild-type HEXIM1, both mutants were unable to repress Tat as well as Gal4-CycT activities, albeit they were expressed at levels comparable to the wild-type protein. Collectively, the results presented in figures 3 and 4 strongly suggest that HEXIM1-mediated inhibition of Tat activity requires interaction with P-TEFb as well as binding to 7SK snRNA.

### P-TEFb activity in the presence of enhanced expression of HEXIM1

**Figure 5:**
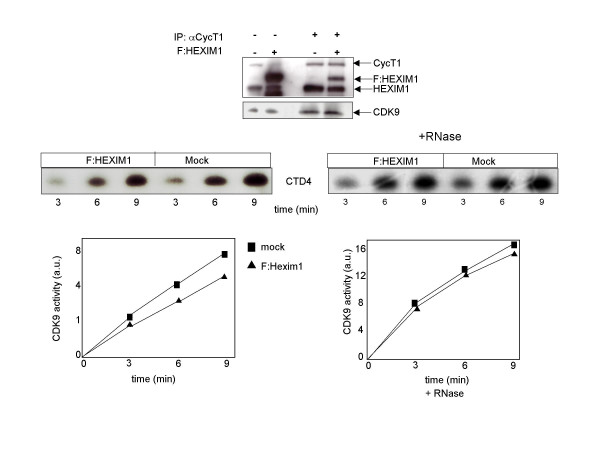
P-TEFb activity in F:HEXIM1 transfected cells. Human 293 cells were transfected with 100 ng of F:HEXIM1 and cell extracts were prepared from mock and F:HEXIM1 expressing cells at 48 hr after transfection. Cell extracts were immunoprecipitated with anti-cycT1 antisera. The relative amounts of immunopreicipitated cyclinT1, CDK9 and HEXIM1 were quantitated by immunoblotting. Samples were treated or not treated with RNase, as indicated. Kinase assays were performed using a CTD4 peptide and ^32^P incorporation was quantified in arbitrary units and plotted versus time (min). This experiment was performed four times with similar results. A typical experiment is shown.

Next we sought to determine whether enhanced expression of HEXIM1 might directly affect the P-TEFb activity. 293 cells were transfected with F:HEXIM1 and cellular extracts from mock and transfected cells were prepared. P-TEFb activity was assayed using as substrate the CTD4 peptide consisting of four consensus repeats of the RNAPII CTD, and time-course kinase assays were performed [15]. Briefly, P-TEFb and its associated factors were affinity purified with anti-CycT1 antibody from mock and F:HEXIM1 transfected cell extracts. Immunoprecipitates were analyzed by immunoblotting for evaluation of CDK9, cyclin T1 and HEXIM1 proteins, respectively. The immunoprecipitates were then treated or not treated with RNase A (Fig. 5). The RNase treatment will degrade the 7SK snRNA thereby relieving the P-TEFb inhibition by HEXIM1/7SK snRNP. In fact, samples treated with RNase showed a robust increase in kinase activity compared those not treated with RNase, indicating that 7SK snRNA had been effectively degraded. We found that the kinase activities of samples that were treated with RNase were quantitatively the same in both mock and F:HEXIM1 transfected extracts indicating equal amounts total of P-TEFb in both samples. A modest, but reproducible reduction of P-TEFb kinase activity (2-fold) was observed in extracts from F-HEXIM1 transfected cells. Altogether, these results demonstrated that over-expression of HEXIM1 resulted in a modest reduction of P-TEFb activity, thus the inhibition of Tat activity is unlikely due to a global reduction of cellular P-TEFb activity.

**Figure 6:**
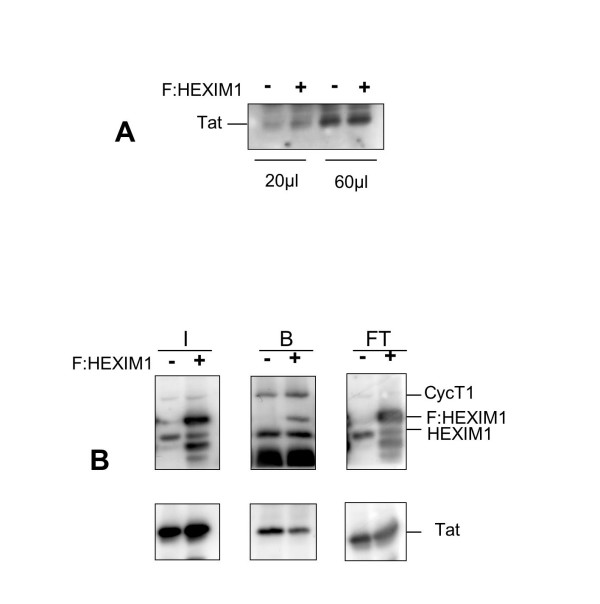
Tat-CyclinT1 binding in the presence of HEXIM1. Panel A. 293 cells were transfected with 50 ng of pSV-Tat in the presence or absence of F:HEXIM1 (100 ng) as indicated and at 48 hrs after transfection cell extracts were probe by Western blotting with anti-Tat. For accurate comparison increasing amounts of material (μl) were loaded on the gels. Panel B. 293 cells were transfected as in Panel A, and cell extracts were immunoprecipitated with anti-CycT1. Immunocomplexes were analyzed on Western blots as indicated. I, input, B; bound, FT; flow through. This experiment was performed two times with similar results.

To further investigate the mechanism of inhibition of Tat-mediated transcription by HEXIM1, we tested the relative levels of transfected Tat protein in the presence of F:HEXIM1. We found that ectopic expression of HEXIM1 did not affected Tat expression (Figure 6A). Next, we sought to determine whether exogenous expression of HEXIM1 might result in a decrease in Tat-bound CycT1. To this end 293 cells were transfected with pSV-Tat in the presence or absence of F-HEXIM1 using the same transfection conditions used in the Luciferase assays. Cells extracts were immunoprecipitated with CycT1 antibody and the immunoprecipitates were analyzed by immunoblotting for evaluation of Tat, CycT1 and HEXIM1 proteins, respectively. In two different experiments we found a modest, but reproducible decrease in Tat-bound cyclin T1 (Fig. 6B). Thus, it appears that exogenous expression of HEXIM1 results in a decrease of Tat-bound P-TEFb.

## Discussion

Several lines of evidence have suggested that Tat function is largely dependent upon the physical and functional interaction with the cellular transcription factor P-TEFb. The recruitment of P-TEFb to the LTR, involves the formation of the Tat-P-TEFb complex which efficiently binds TAR, allowing CDK9 to phosphorylate the CTD of RNAPII, thereby, enhances processivity of the polymerase to produce full-length mRNAs [6-10]. Two different P-TEFb complexes exist in vivo. The core active P-TEFb comprises two subunits, the catalytic CDK9 and a regulatory partner cyclin T, and a larger inactive P-TEFb complex comprised by CDK9, cyclin T, HEXIM1 protein and the 7SK snRNA [11-17]. The relative presence of core and inactive P-TEFb complexes changes rapidly in vivo [11]. We have previously shown that the presence of HEXIM1/7SK snRNA in P-TEFb complexes prevents Tat binding to P-TEFb [13]. Since the association between 7SK RNA/HEXIM1 and P-TEFb competes with binding of Tat to cyclinT1, we have speculated that the TAR RNA/Tat system may compete with the cellular 7SK snRNA/HEXIM1 system [13]. Accordingly, it has been shown that over-expression of HEXIM1 represses Tat function [14,19] We show here that HEXIM1 inhibits Tat function, while expression of 7SK snRNA does not influence Tat activity. It is pertinent to note that 7SK RNA is an abundant snRNA [23], and it is unlikely that 7SK might be rate-limiting for the assembly of the inactive P-TEFb complex.

We have delineated important structural domains of HEXIM1 required for repression of Tat. First, we found that the C-terminal region is required for inhibition. Previous findings indicated that the C-terminal region of HEXIM1 is involved in binding with cyclinT1 as well as for homo and hetero-dimerization with HEXIM2 [15,18,19]. Second, point mutations in the evolutionarily conserved motif PYNT (aa 202–205) abolished inhibition. It has recently shown a critical role of threonine 205 in P-TEFb binding [15]. Moreover, deletion mutants unable to bind P-TEFb failed to repress Tat (Figure 3). Therefore, it appears that HEXIM1 inhibition is strictly dependent upon the integrity of the protein to interact with P-TEFb. Third, a point mutant in the central part of HEXIM1 (KHRR motif aa 152–155) strongly affects Tat repression. Since this basic motif has been previously shown as the 7SK snRNA recognition motif [15], we conclude that interaction between HEXIM1 and 7SK snRNA is required for Tat repression. Collectively, these findings strongly suggested that HEXIM1-mediated inhibition of Tat required the formation of the P-TEFb/HEXIM1/7SK complex.

We determined that enhanced expression of HEXIM1 resulted in a modest inhibition (2-fold) of P-TEFb activity in vivo. Thus, HEXIM1-mediated inhibition of Tat activity is unlikely due to a global inhibition of P-TEFb activity. Moreover, we found that basal transcription from the LTR sequences was largely unaffected by over-expression of HEXIM1. Finally, ectopic expression of this protein does not have significant effects on TBP-mediated basal transcription. Thus, it appears that P-TEFb is specifically required for Tat-dependent HIV LTR transcription. Our results differ somewhat from those obtained in the Zhou lab who found that exogenous expression of HEXIM1 affects both basal as well as Tat-induced transcription [13]. These apparent discrepancies are possible due to different transfection conditions in which the relative amounts of the over-expressed exogenous proteins are likely different. We found that Tat expression which is under the control of SV40 promoter remains largely unaffected by co-expression of HEXIM. Our findings suggest a dedicated role of P-TEFb in Tat activity. Recent studies point to a specific role of P-TEFb for certain promoters. It has recently found that P-TEFb is recruited to the IL-8 but not to the IkBα promoter [23], and it also represses transcription of regulators such as the nuclear receptor coactivator, PGC-1, in cardiac myocytes [24]. The specific HEXIM-mediated inhibition of Tat activity underlines the pivotal role of P-TEFb in the HIV LTR transcription.

The repression exerted by the HEXIM1 protein is likely the results of a competition between Tat and HEXIM1 in binding the P-TEFb. Since Tat binds only to the active P-TEFb complex, it has been suggested that Tat might trap the active form of P-TEFb as the PTEFb/7SK RNA/HEXIM1 complex appears to undergo continuous formation and disruption *in vivo*. In this scenario over expression of HEXIM1 may counteract the binding of Tat to P-TEFb, through a competitive association between the ectopic expressed HEXIM1 and P-TEFb. Accordingly, we found that exogenous expression of HEXIM1 results in a small but detectable reduction in Tat-bound- P-TEFb. Our co-immunoprecipitation results are consistent with recent findings showing a mutually exclusive interaction of HEXIM1 and Tat with cyclinT1 using recombinant purified proteins [25]. Because Tat and HEXIM1 interact with the cyclin-box region of cyclinT1, it is plausible if not likely, that the mutually exclusive interaction of these two molecules with cyclinT1 is due to binding to the same domain or to a sterical hindrance. However, these studies have been performed in vitro in the absence of 7SK snRNA.

The results reported here along with previous findings strongly suggest the crucial role of 7SK in the interaction between HEXIM1 and cyclinT1. In fact, HEXIM1 ILAA mutant does not associate with 7SK in vivo and in vitro, and co-immuprecipitation of cyclinT1 and 7SK RNA was markedly reduced with ILAA mutant compared to wild type [15]. Finally, as shown here ILAA mutant failed to repress Tat activity, suggesting an important role of HEXIM1/7SK interaction in Tat inhibition. Thus, association between HEXIM1 and 7SK snRNA appears an important determinant for Tat inhibition. Future in vitro and in vivo interaction studies, in the presence of 7SK snRNA may be instrumental to elucidate the role of 7SK/HEXIM1 complex in Tat activity.

## Conclusion

The studies described in this provides further support to the pivotal role of P-TEFb for the optimal transcription Tat activity and highlight the importance of the P-TEFb cellular co-factors HEXIM1/7SK snRNA complex in Tat activity.

## Methods

### Tissue culture and transfections

Human 293 and rodent CHO cells were grown at 37°C in Dulbecco's modified Eagle's medium (DMEM) supplemented with 10% fetal calf serum (Gibco, Life Technologies). Subconfluent cell cultures were transfected cell cultures were transfected by a liposome method (LipofectAMINE reagent; Life Technologies, Inc.) in 2 cm/dish in multiwells, using 100 ng of reporter DNA and different amounts of activator plasmid DNA as indicated in the text and 20 ng of Renilla luciferase expression plasmid (pRL-CMV, Promega) for normalization of transfections efficiencies. Cells were harvested 48 h after DNA transfections, and cellular extracts were assayed for luciferase activity using Dual-Luciferase Reporter assay (Promega) according to the manufacturer's instructions. The experimental reporter luciferase activity was normalized to transfection efficiency as measured by the activity deriving from pRL-CMV.

### Plasmids

The G5HIV-Luc contained the HIV-1 LTR sequences from -83 to +82 of LTR driven the Luc gene with 5 GAL4 DNA-binding sites inserted at -83. The pSV-Tat, GAL4-TBP, GAL4-CycT1, have been described [20]. 7SK snRNA plasmid was kindly provided by S. Murphy [22]. All Flag-taggeted HEXIM1 and HEXIM2 expression vectors were constructed by insertion of the corresponding cDNA regions into the EcoRV site of p3xFlag-CMV10 vector (Clontech). Description of the deletion and point HEXIM1 mutants have been described previously [15]. Full description of the expression vectors used in this work is available upon request.

### Western blotting and antibodies

Cells were lysed in ice-chilled buffer A (10 mM HEPES pH 7.9, 1.5 mM MgCl_2_, 10 mM KCl, 200 mM NaCl, 0.2 mM EDTA), supplemented with 1 mM dithiothreitol, 40 U/ml of RNasin (Promega), protease inhibitor cocktail (P-8340; Sigma), and 0.5 % Nonidet P-40. Lysates were vortexed and incubated for 20 min on ice and clarified by centrifugations. Western blottings were performed using the following antibodies: the rabbit polyclonal anti-HEXIM1 (C4) has been previously described (6); anti-FLAG M2 Monoclonal Antibody (Sigma), goat polyclonal anti-CycT1 (T-18), rabbit polyclonal anti-CDK9 (H-169) from Santa Cruz, anti-Tat (NIH AIDS Research Reagent Program). Binding was visualized by enhanced chemiluminescence (ECL-plus Kit, Amersham Biosciences).

### Co-immunoprecipitation and kinase assay

293 cells were transfected with pSV-Tat in the presence or absence of F:HEXIM1 and cell extracts were prepared at 48 hrs after transfection. CycT1 was immunopurified from cell extracts (1 mg) using anti-CycT1 (H-245) (sc-10750, Santa Cruz). Input, immunoprecipited and flow through materials were used in western blottings using anti-cycT1, anti-HEXIM1 and anti-Tat, respectively. For kinase assays 293 cells were transfected with F:HEXIM1 and after 48 hr P-TEFb complex was immunopurified from cell extracts (1 mg) using anti-CycT1 (H-245) (sc-10750, Santa Cruz) as previously described [13,15]. Briefly, whole cell extracts from mock and F:HEXIM1 transfected 293 cells were used in immunoprecipitations together with 40μl of slurry beads (protein G-Sepharose 4 Fast Flow, Amersham Biosciences) pre-adsorbed with anti-CycT1 and the interactions were carried out in buffer A for one hour at 4°C on a wheel. After extensive washes one half of the immunopurified materials was used in western blotting to ensure the presence of equal amounts of CDK9, HEXIM1 and CycT1, respectively. The remaining material was suspended and stirred at room temperature and split in two equal aliquots. One of the aliquot was treated with 10U of RNase A for 15 min at 30°C. Samples treated or not with RNase were stirred at room temperature for three minutes in 65 μl of buffer A containing [γ-^32^P]ATP (0,1 μCi/μl), 40 mM ATP, 0,1 μg/ml (YSPTSPS)_4 _peptide CTD4 (6, 8) and RNasin (40 U/ml). Aliquots (20 μl) of the suspension were mixed with SDS-PAGE loading buffer at intervals of three minutes to stop the reaction. The phosphorylated CTD4 substrate was separated on a 15% SDS-PAGE and visualized by radiography. Incorporation of [^32^P] into CTD peptide was quantified on a Bio-Rad phosphoimager.

## Competing interests

The author(s) declare that they have no competing interests.

## Authors' contributions

AF carried out the transfection studies and plasmid construction. FV performed studies using the HEXIM1 point mutants. GN carried out the kinase experiments. AAM isolated and constructed the HEXIM2 expression vector. BM and OB participated on discussion of results and drafting the manuscript. LL designed this study and edited the manuscript.
